# *Fusarium graminearum* Colors and Deoxynivalenol Synthesis at Different Water Activity

**DOI:** 10.3390/foods8010007

**Published:** 2018-12-23

**Authors:** Edgar Cambaza, Shigenobu Koseki, Shuso Kawamura

**Affiliations:** 1 Laboratory of Food Process Engineering, Graduate School of Agriculture, Hokkaido University, Sapporo, Hokkaido 060-0808, Japan; koseki@bpe.agr.hokudai.ac.jp (S.K.); shuso@bpe.agr.hokudai.ac.jp (S.K.); 2 Department of Biological Sciences, Faculty of Sciences, Eduardo Mondlane University, Av. Julius Nyerere, 3453 Maputo, Mozambique

**Keywords:** *Fusarium graminearum*, deoxynivalenol, RGB, water activity

## Abstract

Deoxynivalenol (DON) is a well-known mycotoxin, responsible for outbreaks of gastrointestinal disorders in Japan. *Fusarium graminearum*, a parasite of cereal crops, produces this toxin and this is one of the reasons why it is important to understand its metabolism. It is possible to predict the mold’s color change and the quantity of DON synthesized throughout its lifecycle. Furthermore, a_w_ has been found to affect the amount of DON. This study aimed to analyze the potential of *F. graminearum* surface color as a predictor of DON concentration at a_w_ = 0.94, 0.97, and 0.99. Thus, 36 specimens were incubated at 25 °C, 12 at each a_w_. After 4, 8, 12, and 16 days, three specimens from each a_w_ were collected for color analysis and DON quantification. For color analysis, photos were taken and red, green and blue (RGB) channels were measured on ImageJ software. DON was quantified through liquid chromatography (HPLC). Color changes were only observed at a_w_ = 0.99 because at lower a_w_ the molds presented high growth of white mycelium. Yet, DON increased in all cases. It was only possible to relate the colors with DON concentration at a_w_ = 0.99, where they presented inverse proportionality.

## 1. Introduction

**Figure 1:**
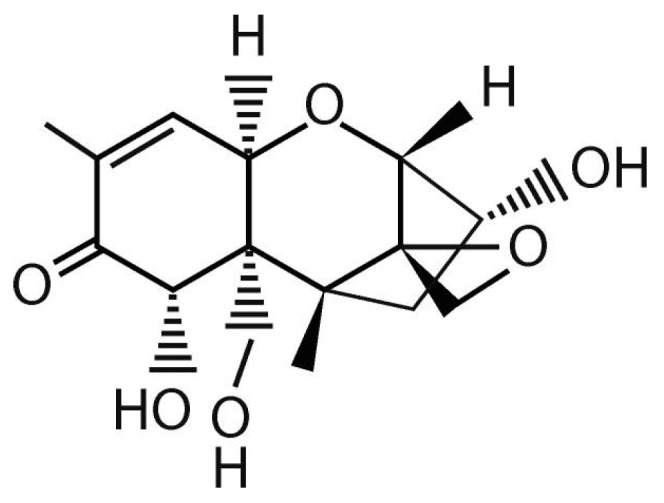
Deoxynivalenol (DON) structure. Based on Sobrova, et al. [2].

Mycotoxin studies have been gaining prominence since the second half of the 20th century, and deoxynivalenol (DON) or vomitoxin (H_15_O_20_O_6_, Figure 1) is among the most well-known among these toxins [1]. In its physical form DON form colorless fine needles, it is soluble in polar organic solvents and water and its melting point is 151–153 °C [2]. DON belongs to the class of trichothecenes and causes nausea, vomiting, diarrhea, abdominal pain, headache, dizziness, and fever [1,2]. The World Health Organization considers DON as a teratogenic and immunosuppressive neurotoxin [3].

DON highly affects the cost of commodities as it increases the use of fungicide and the expense to screen them for toxicity [4]. According to Schmale, III and Munkvold [5], DON is responsible for losses of approximately $655 million per year in the United States, mostly in wheat. The toxin was also identified as the cause of at least eight outbreaks of intoxication in Japan, including two cases in the Hokkaido prefecture. The toxin is among the natural contaminants described by the country’s Ministry of Health, Labor, and Welfare as a potential threat for public health [6]. DON is frequently found in corn, wheat, oats, barley, rice, and other grains and derivatives [2].

Water activity (a_w_) is among the environmental factors with impact on the quantity of DON produced by *Fusarium graminearum* (teleomorph: *Gibberella zeae*) [7,8,9]. Though there are still some inconsistences on how they are related, increased a_w_ seems to favor higher DON production [10]. According to Leplat, et al. [11], at 25 °C *F. graminearum* a_w_ tolerance is between 0.9 and 0.995, being the optimal range between 0.95 and 0.995 (higher a_w_ values are not mentioned). Regarding DON, the same authors said that deoxynivalenol has been only detected between a_w_ of 0.95 and 0.995. Furthermore, a_w_ is frequently used in models to predict mycotoxin concentration in vitro, together with temperature and other variables such as concentration of nutrients or fungicides [12]. Still, there is need to further explore, expand and propagate the current knowledge on how a_w_ affects DON synthesis by *F. graminearum*.

The RGB (red, green and blue) components of *F. graminearum* surface color were recently found to exhibit predictable changes over time [13], and this feature is desirable as an alternative to size measurement to estimate the mold’s maturity because size is highly dependent limitations, such as the borders of a Petri dish and it does not provide much information about the metabolism [13]. Since both DON concentration [8,14] and surface color [13] are predictable for *F. graminearum* over time, it is reasonable to admit the possibility that both can be related at certain degree. Furthermore, surface color and toxin concentration are manifestations of the mold’s state of maturity [13,15].

This study aims to demonstrate that *F. graminearum* surface color can be used to predict how much DON the fungus produces taking a_w_ in consideration. These analyses will consubstantiate the idea that color is a viable alternative to size in in vitro mold growth studies.

## 2. Materials and Methods

### 2.1. Mold Isolate

This study used an *F. graminearum* isolate from the Catalogue of the Japan Collection of Microorganisms (JCM). It is registered as the teleomorph *Giberella zeae* (Schwabe) Petch, strain TH-5, isolated by Sugiura [16] from rice stubble in Hirosaki, Aomori Prefecture, Japan. It is a known producer of deoxynivalenol, 15-acetyldeoxinivalenol, and zearalenone [17].

### 2.2. Experimental Procedure

#### 2.2.1. Incubation and RGB Determination

**Figure 2:**
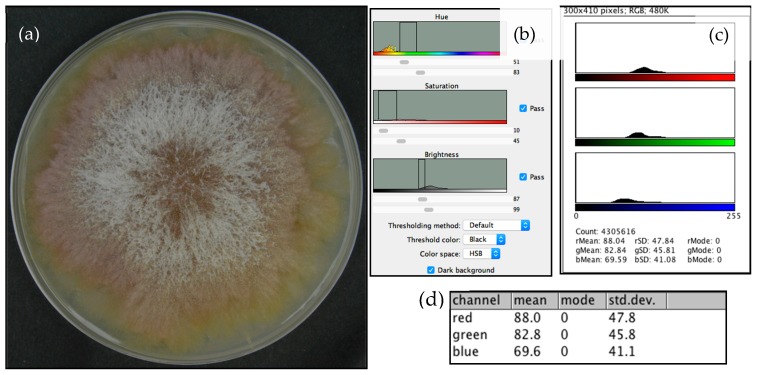
Process of *F. graminearum* color analysis using ImageJ: (**a**) sample photo of the mold; (**b**) ImageJ panel used to remove the background by filtering colors; (**c**) color measurement panel; and (**d**) color measurement table.

Despite the previous of Leplat, et al. [11], a_w_ of 0.94 was found to be the lowest at which the mold grew on yeast extract agar (YEA). Thus, 36 specimens of *F. graminearum* were grown at 25 °C on yeast extract agar at three water activity (a_w_) settings experimentally prepared using glycerol: 0.94, 0.97, and 0.99. From the fourth incubation day, three replicates per a_w_ were taken for DON quantification. Before the extraction, the fungi were photographed in a black bucket, vertically from 30 cm above. The camera model was Nikon D3200 with a lens DX SWM VR (Nikon Corporation, Tokyo, Japan), and it was used without flash or any automation affecting illumination. The only source of light was a round LED attached to the bucket’s lid. The photos were then processed using the method described by Cambaza, et al. [13] (Figure 2) on ImageJ software (FIJI edition, National Institutes of Health, Bethesda, MD, USA), developed by the National Institutes of Health and the Laboratory for Optical and Computational Instrumentation (LOCI, University of Wisconsin) [18]. ImageJ allowed the determination of average intensities of the RGB components from the photos.

The analysis considered only the fungal surface, excluding any background including the plate borders or agar. In the end, the variables to analyze were the incubation time (in days), a_w_ and the RGB parameters, converted from the eight-bit notation (0−255) to the arithmetic index (0.0−1.0).

#### 2.2.2. Extraction and High-Performance Liquid Chromatography (HPLC)

For extraction, each sample was mixed with 100 mL of water:acetonitrile (84:16) and blended in a Seward Stomacher 400 machine (Seward Ltd., Singapore). Approximately 15 mL of the filtered extract was transferred to a tube and 2 mL of this filtrate were purified using Supel™ TOX DON cartridges [19]. These cartridges eliminate undesirable fat, pigment, and carbohydrate and retain large molecules. HPLC was run through a Jasco CrestPak C18T-5 affinity column using (a) water:acetonitrile:methanol (92:4:4) and (b) acetonitrile as a mobile phase with a flow rate of 0.2 mL/min at 35 °C and detection set for ultraviolet light at 220 nm. DON peak consistently appeared at 8 min.

### 2.3. Statistical Analysis

The statistical analysis was performed on JASP 0.9 (The JASP Team, Amsterdam, The Netherlands), Jamovi 0.9 (Jamovi Project, Amsterdam, The Netherlands) and Microsoft Excel (Version 14.5.8, Microsoft, Redmond, Washington, WA, USA). All the hypotheses tested were carried out with α = 0.05. The distribution of intensities of red, green, and blue were compared through analysis of covariance (ANCOVA) to find if their differences were significant. Then, the relationships between the colors were analyzed through a scatter plot matrix. Subsequently, the focus oriented towards each color. For each, a Kruskal-Wallis test determined if the distribution of color intensity between the samples grown at distinct a_w_ presented significant differences. The final step analyzed the impact of a_w_ on the pigmentation and DON concentration.

## 3. Results

**Figure 3:**
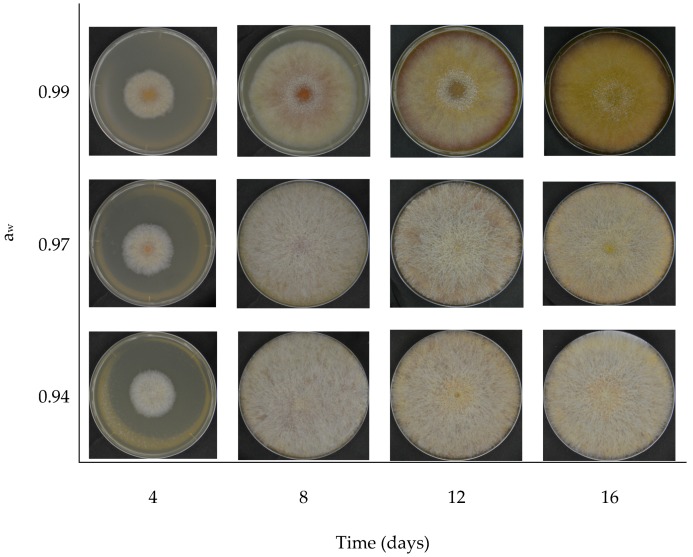
Surface color of *F. graminearum* grown at different a_w_ for 16 days.

All the specimens grew throughout the 16 days and measurements were successfully carried out. The ones grown at distinct a_w_ presented notable visual differences in color and texture (Figure 3), particularly the molds grown at a_w_ = 0.99 in relation to the others. However, all specimens were mostly similar up to the fourth day, developed into a white mycelium with a diameter of approximately 3 cm with a yellow spot at the center, resembling a fried egg. The central spot was less visible in the specimens grown at a_w_ = 0.94 and it was increasingly noticeable as the water activity increased.

The specimens grown at a_w_ of 0.94 and 0.97 showed high rate of mycelial growth up to day 8, covering the entire plate with its radially dispersed hairy whitish surface, and seemed to remain unchanged until the end of the experiment. In some cases, the mycelial growth was immense, touching the Petri dish’s lid. However, the molds incubated at a_w_ of 0.99 did not produce as much mycelial growth and exhibited more clearly visible concentric areas with distinct colors, each with notable changes from one measurement to the following. Its central spot changed to reddish, brown and, finally, pale, seemingly because of some white mycelial growth on top. Its borders developed a wine red tone and the surface became increasingly yellow. These observations suggest that *F. graminearum* surface color is highly sensitive to a_w_, and a_w_ reduction promotes mycelial growth, possibly as a stress factor.

Table 1 confirms the impact of a_w_ on the mold’s color, especially the green and blue components (*p*_ANCOVA_ < 0.05). Red color did not seem to be significantly affected by a_w_, even after Tukey’s post hoc comparisons. Green and blue showed exactly the same profile of significance considering the different a_w_, though green showed highest levels of discrepancies in all cases. The overall differences, measured through ANCOVA were significant. Regarding the post hoc comparisons, the significant differences occurred between the specimens incubated at a_w_ of 0.99 and the others. These observations are consistent with the visual analysis in which a_w_ reduction drastically affects *F. graminearum* color pattern.

**Figure 4:**
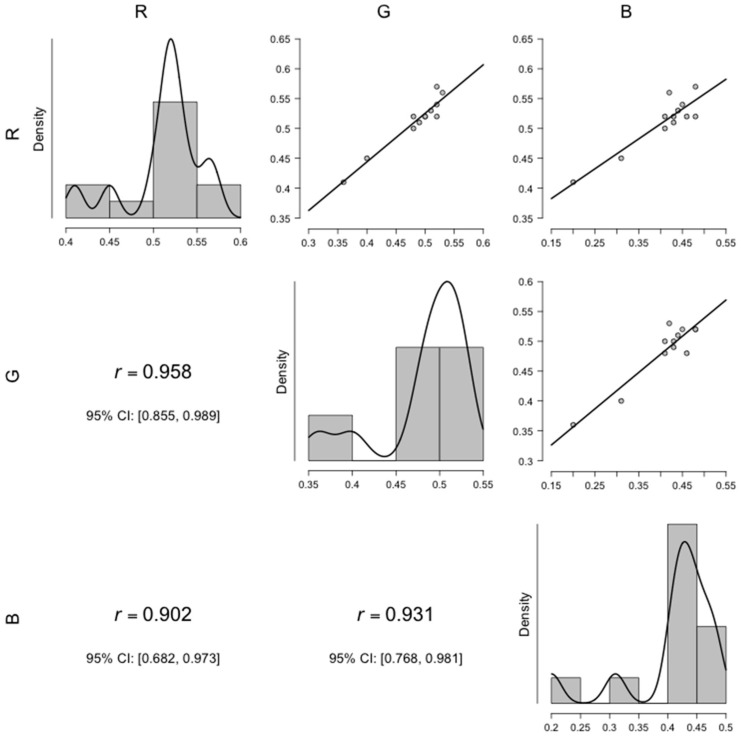
Pearson’s correlations between the RGB components. The diagonal charts show the intensity of the colors. CI = confidence interval; *r* = Pearson’s coefficient.

Despite of the differences between the colors at distinct a_w_, all three RGB components seemed highly correlated (Figure 4), with Pearson’s correlation *r* above 0.9.

The data suggest direct relationships between them, and all colors showed considerably high density of their lighter shades. Red and green were the most strongly correlated, followed by blue and green. Thus, even though the red component seemed to be consistently the same through at different a_w_, unlike the others, its slight variations presented a similar profile to the ones exhibited by the green and blue channels.

**Figure 5:**
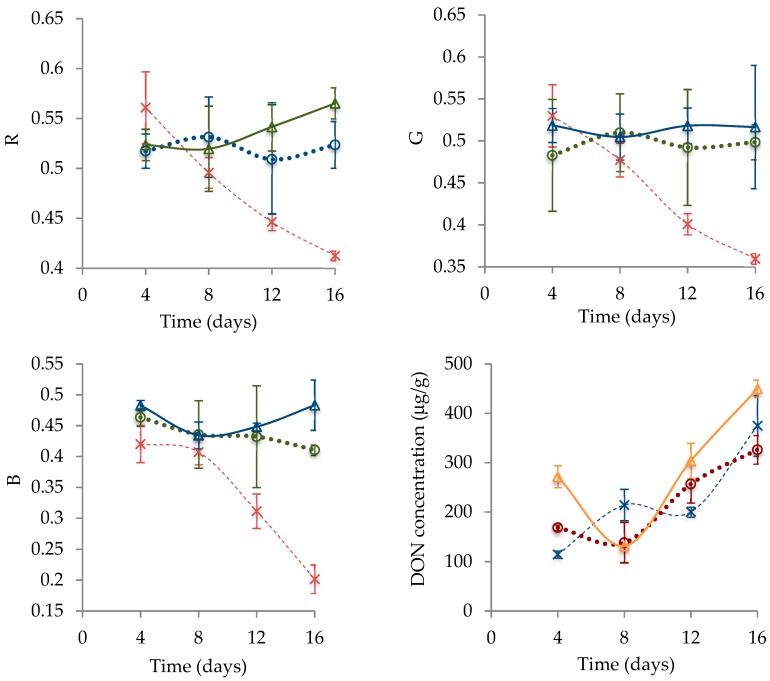
Variation of RGB components and DON concentration over time under different a_w_. Note: R = red; G = green; B = blue; a_w_ = 0.94 (Δ); a_w_ = 0.97 (O); a_w_ = 0.99 (×).

Figure 5 shows the variations in RGB components and DON concentration over time considering the different a_w_ settings. The colors seemed to exhibit very similar patterns of variation over time. The specimens grown at a_w_ = 0.99 decreased in color intensity (all RGB channels) while the others apparently remained constant and considerably high. This is consistent with the photos, where the lowest a_w_ incubation setting resulted in predominantly whitish surface during virtually the entire experiment. At a_w_ = 0.99, the best simple algebraic representations were *y* = 0.0005*x*^2^ − 0.0222*x* + 0.6416 for red, *y* = 0.0002*x*^3^ − 0.0044*x*^2^ + 0.023*x* + 0.4992 for green and *y* = 0.0002*x*^3^ − 0.0068*x*^2^ + 0.0588*x* + 0.2831 for blue, all with *R*^2^ = 1, assuming *x* as time in days and *y* as the RGB component within the scale 0 to 1. DON concentration seemed to increase in general for all a_w_ settings, though there are incidental cases of reduction.

It is hard to explain why there are reductions because the toxin is expected to accumulate over time but it might have been due to cross-contamination in the column, possibly because the time initially set to clean it up after measurement (10 min) might not have been enough. It was then set to 20 min just to ensure that the column could be properly cleaned.

An analysis of covariance (ANCOVA) shows no significant differences between DON concentrations (*p* = 0.347) of samples incubated at different a_w_. Since the colors change their pattern of variation when the molds are subjected to distinct a_w_, but it does not happen to DON: The high superficial mycelial growth in the specimens at lower a_w_, the fungus seems to keep the ability to produce the toxin even when there is higher mycelial growth. It is possible that the layer of whitish hyphae is masking an inferior highly pigmented layer in the specimens grown at lower a_w_. The bottom-line is perhaps the fact that lowering a_w_ caused the white mycelium to remain abundant throughout the experiment while DON kept accumulating.

**Figure 6:**
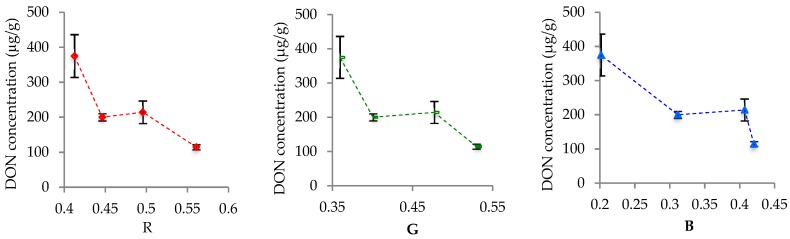
Relationship between color variation and DON concentration at a_w_ = 0.99.

Only the RGB channels at a_w_ = 0.99 could be used as independent variables to plot with DON concentration (Figure 6) because colors did not change significantly at lower a_w_.

All colors decreased in value, oscillating once. The major differences between the colors seemed to be the wideness and position of their dominium (range of abscissae). Considering *x* as the RGB channel and y as the DON concentration, as one observed from the origin of *x* towards 1, blue presented the lowest values but also the widest range, followed by green with intermediate values and range, and red. The considerably narrower range of the red component may explain why it did not present significant differences across a_w_.

## 4. Discussion

In summary, a_w_ had a major impact on *F. graminearum* surface color. Up to day 4, all the specimens were mostly similar in appearance, with a yellowish center surrounded by a whitish mycelium, resembling a fried egg. However, a reduction in a_w_ seemed to promote *F. graminearum* mycelial growth, masking its conidial pigmentation. As consequence, the specimens grown at a_w_ = 0.97 and 0.94 remained whitish throughout the entire experimental period, with RGB channels presenting no significant variations, unlike the molds grown at a_w_ = 0.99. In any case, the RGB components appeared highly correlated, with Pearson’s coefficient *r* > 0.9 when the colors were considered two at a time. Yet, only green and blue components exhibited significant variations, even though all colors had the same pattern of variation. The significant differences in green and blue were only between the samples incubated at a_w_ = 0.99 and the others, and this supports the previous observations from the photos. The highest a_w_ was marked by a reduction of RGB components, all fit to polynomial functions. The lowest a_w_ settings presented notably constant trends. Still, DON concentration increased in all a_w_ settings, independently of the surface color. Thus, only the highest a_w_ was considered to build graphs relating DON concentration with color variation. They seemed inversely proportional if colors represented as the abscissae and DON concentration the ordinate. As one moved from the origin of abscissae, the blue, green and red ranges appeared (overlapping), each narrower than the previous but all with the same shape.

Water activity is among several factors affecting the pigmentation of *F. graminearum* [13,20,21,22]. The way it affects can be very complex because the mold’s surface color results from the combination of several different pigments, some with quite different chemical properties [21,23,24,25,26]. For instance, a_w_ partially affects its chromatic attributes of the polyketide aurofusarin, perhaps the most influential pigment, notable for its yellow and red coloration [27]. Yet, the color differences appeared more associated with the increased growth of white mycelium on top of the mold, covering the entire dish, rather than caused by changes in nature or quantity of pigments. The simple fact that lower a_w_ stimulated higher mycelial growth might look counterintuitive and also contradicts previous observations [8,28], but it makes some sense that the shortage of water leads the fungus to expand its hyphae in search for new sources [29]. The initial similarity between the specimens grown at distinct a_w_ perhaps occurred because the molds were very small and the shortage of water was not yet impacting the mycelia. As they grew, the ones grown at lower pH experienced early exhaustion of water and seemed to react by expanding hyphae to all directions including upwards. Furthermore, during day 4 they were still at exponential growth [13], with minor differentiation.

The fact that all RGB components were highly correlated supports the idea that a small set of pigments with similar colors is producing them. Otherwise, one should expect each RGB component to exhibit its own pattern of variation if there were a wide variety of pigments with different colors, especially if the pigments were chemically diverse. The literature identifies aurofusarin [30], as already mentioned, and the carotenoid neurosporaxanthin [31,32] as the major pigments influencing the surface color of *F. graminearum*. They are both yellow, though slightly different. The former is frequently described as “golden yellow”, though its hue varies to orange and wine red as it changes to derivatives [21,23,30,33], and neurosporaxanthin was described as “orange-yellowish” [34], just like most carotenoids. There are also the polyketide rubrofusarin [21] and the carotenoid torulene [25], both red but not as abundant as the previously mentioned. There are more pigments but they have minor influence on the overall color [34] or only during differentiation [35,36]. As bottom-line, the only pigments actually influencing the color have similar or close-related hue ranging from golden yellow to wine red. It is worth mentioning that the polyketides (aurofusarin and rubrofusarin) are highly bioactive and possibly essential part of the competitive saprophytic ability (CSA) of *F. graminearum* [33,37], the carotenoids are not likely and the latter tend to respond mostly to light rather than nutrients [24], certainly except in extreme cases of shortage of some nutrient essential for synthesis of such pigments. Thus, the polyketides, especially aurofusarin, appeared to be key pigments contributing *F. graminearum* surface color variation in the current experiment.

A previous experiment had already shown that all RGB components exhibit similar pattern of variation, consistent with third-degree functions [13]. It is not clear why the red component did not show significant variation (α = 0.05) while the other colors did, but it might be related to the nature of the most abundant pigments [15]. Perhaps red pigments such as rubrofusarin and torulene, especially the latter, do not change their colors throughout the mold’s lifecycle, contributing to this “resistance” to change. However, both pigments probably suffer a considerable reduction because the former is an intermediate of aurofusarin synthesis [38] and the latter is a precursor of neurosporaxanthin [39]. Yet, both pigments have been found in *F. graminearum* matrix, even when the others are present [21,39], from which one can imply the existence of chemical equilibrium between them. It this case, it is still possible that rubrofusarin and torulene contribute to the endurance of the red component.

RGB values were expected to decrease throughout the experiment, especially for blue, followed by green and, finally, red. According to a previous experiment [13], this RGB reduction corresponds to the darkening process as the fungus grows towards the stationary growth phase. It surely does not apply in the cases in which the fungi were covered with white mycelia because it did not allow the pigments to be visible. In the cases where the color changed, the variation of RGB components was passible of representation through polynomial curves, and this was also observed in the aforementioned experiment.

All samples showed overall increased DON concentration over time, not mattering if there was high mycelial growth or not on the surface. There is some counterintuitive reduction for the samples grown at a_w_ = 0.94 and 0.97 between days 4 and 8, but it was likely due to fluctuations in the results. Indeed, the ANCOVA test (*p* = 0.347) suggested that the differences between the DON concentrations at different a_w_ were not significant. This result contrasts with some found in the literature showing significant differences between DON concentrations at distinct a_w_ [7,8,9]. Though it is difficult to know why these results were counterintuitive, it might have been due to chemical, genetic (distinct strains) or nutritional differences [10]. All other experiments were performed with irradiated wheat, while the current was carried out with YEA. The latter is highly nutritive [40] and this perhaps attenuated the stress caused by a_w_ differences. Furthermore, Sorensen and Sondergaard [41] demonstrated that even different yeast extracts influence DON concentration. Anyway, the studies on wheat showed similar trends disregarding a_w_, and it is intuitive that DON tends to accumulate over time because mycotoxins are very stable and the fungi do not metabolize them [29,42]. 

DON concentration seems to have similar relationship with all RGB components at a_w_ = 0.94 and it will facilitate use colors as an alternative to size in DON analysis at this a_w_. This subsidizes the previous study demonstrating that *F. graminearum* color variation is predictable throughout its life cycle [13]. There is also evidence that biosynthesis of the pigment aurofusarin is related to DON production as histone H3 lysine 4 methylation (H3K4me) is crucial in the transcription of genes for synthesis of both compounds [15]. Yet, the relationship between the pigment and DON still requires further biochemical and genetic investigation. In any case, as far as it showed, *F. graminearum* surface color can be used in microbiological studies to predict DON concentration at a_w_ = 0.99, but it does not seem practical for lower a_w_.

## 5. Conclusions

The current experiment suggested that all RGB channels obtained from photos of *F. graminearum* are correlated and can be used to predict DON concentration produced by the fungus at a_w_ = 0.99. However, the colors were not effective predictors at a_w_ = 0.97 and 0.94 because these conditions appeared to stimulate the production of white mycelia, barely changing in color. Thus, the results indicate that *F. graminearum* surface color can only be used as predictor of DON concentration at a_w_ as high as 0.99. Future analyses shall find ways to overcome the limitation caused by the superficial white mycelium. One alternative would be to take photos from the bottom or the medium and other, perhaps better, to analyze the color of the *F. graminearum* extract in acetonitrile prior to HPLC because it contains the pigments.

## Figures and Tables

**Table 1:** Color intensity differences between the specimens grown under different water activity.

RGB Channel	*p*_ANCOVA_	a_w_		*Post hoc* Color Comparison				
				Mean Difference	*SE*	*df*	*t*	*p*_Tukey_
R	0.169	0.94	0.97	0.02	0.03	8	0.63	0.809
			0.99	0.06	0.03	8	2.06	0.159
		0.97	0.99	0.04	0.03	8	1.44	0.369
G	0.007	0.94	0.97	0.02	0.01	6	2.22	0.145
			0.99	0.07	0.01	6	8.06	< .001
		0.97	0.99	0.05	0.01	6	5.84	0.003
B	0.02	0.94	0.97	0.03	0.04	8	0.69	0.778
			0.99	0.13	0.04	8	3.43	0.022
		0.97	0.99	0.1	0.04	8	2.74	0.059

R = red; G = green; B = blue; ANCOVA = analysis of covariance; *SE* = standard error; *df* = degrees of freedom; *t* = student’s *t* statistics.
